# Building a KATalogue of acetyllysine targeting and function

**DOI:** 10.1093/bfgp/elv045

**Published:** 2015-10-27

**Authors:** Michael Downey, Kristin Baetz

**Keywords:** KAT, KDAC, acetylation, synthetic lethality, high-content screening, acetylome

## Abstract

Acetylation is a dynamic post-translational modification that is attached to protein substrates by lysine acetyltransferases (KATs) and removed by lysine deacetylases (KDACs). While these enzymes are best characterized as histone modifiers and regulators of gene transcription, work in a number of systems highlights that acetylation is a pervasive modification and suggests a broad scope for KAT and KDAC functions in the cell. As we move beyond generating lists of acetylated proteins, the acetylation field is in dire need of robust tools to connect acetylation and deacetylation machineries to their respective substrates and to dissect the function of individual sites. The *Saccharomyces cerevisiae* model system provides such a toolkit in the context of both tried and true genetic techniques and cutting-edge proteomic and cell imaging methods. Here, we review these methods in the context of their contributions to acetylation research thus far and suggest strategies for addressing lingering questions in the field.

Since the first lysine acetyltransferase (KAT) was identified in 1996 [1], the field of acetylation biology has grown immensely. What was first believed to be a post-translational modification (PTM) limited to histones and the regulation of transcription has in fact turned out be a global and dynamic PTM on par with phosphorylation. In humans alone, acetylation has been identified on thousands of proteins. Given their broad spectrum of substrates, it is not surprising that mutation of KAT and lysine deacetylase (KDAC)-encoding genes have been implicated in a wide variety of human diseases, and therapeutics are increasingly being used or developed to target these enzymes [2]. Although often designed with the intention of modulating specific biological pathways, these drugs are likely to have far-reaching consequences on the cell, changing the acetylation status of hundreds of substrates at a time. Therefore, a major challenge in the field of acetyllysine biology is to characterize the biological functions of KATs and KDACs and identify their substrates.

Given the conservation of KAT and KDAC complexes between yeast and mammalian cells and the fact that acetylated lysines are more conserved between species than non-acetylated lysines [3–5], the toolkits developed for *Saccharomyces cerevisiae* research provide an exceptional means to dissect acetyllysine biology on a large scale. In *S. cerevisiae*, there are 10 KDACs, 8 confirmed KATs and 6 putative KATs (Table 1). Despite over 2000 lysine acetylated proteins that have been identified in yeast large-scale acetylome studies [4, 6, 7], only a handful of KAT/KDAC substrates have been characterized on a functional level (Table 2). Moreover, precious little is known about how these enzymes target their substrates or how such mechanisms are regulated in response to environmental changes to remodel the proteome.

**Table 1:** List of *S. cerevisiae* KATs and KDAC catalytic subunits

**Confirmed KATs**		**Putative KATs**		**Confirmed KDACs**	
Catalytic subunit	Complex	Catalytic subunit	Complex	Catalytic subunit	Complex
Eco1		Hpa2		Hda1	HDA
Elp3	Elongator	Hpa3		Hos1	
Esa1	NuA4; Piccolo NuA4	Lys20		Hos2	Set3C
Gcn5	SAGA; SLIK; ADA	Lys21		Hos3	
Hat1	Hat1-Hat2	Spt10		Hst1	
Rtt109		Taf1	TFIID	Hst2	
Sas2	SAS			Hst3	
Sas3	NuA3			Hst4	
				Rpd3	Rpd3(S); Rpd3(L)
				Sir2	Sir2; RENT

*Note*: Primary references for each KAT and KDAC are further annotated in SGD [8]. For a more detailed overview of *S. cerevisiae* KAT and KDAC complexes, please see [9].

**Table 2:** Non-histone-acetylated yeast proteins for which there exist functional analysis

**Protein**	**Site(s)**	**KAT**	**KDAC**	**Reported function**	**Reference(s)**
Rsc4	K25	Gcn5	???	Chromatin remodelling	[10–12]
Snf2	K1493, K1497	Gcn5	Hst2, Rpd3	Transcriptional regulation	[13]
Ifh1	Multiple	Gcn5	Hst2, Hst1, Sir2	Inhibition of transactivation at RP genes	[14, 15]
Pck1	K514	Esa1	Sir2	Glucose homeostasis	[16]
Sip2	K12, K16, K17, K256	Esa1	Rpd3	Inhibition of Snf1 activity, lifespan and stress control	[17]
Smc3	K112, K113	Eco1	Hos1	Cohesion establishment	[18–23]
Mcd1	K84, K210	Eco1	???	Cohesion establishment	[24]
Mps3	K147, K148, K150	Eco1	???	Sister chromatid cohesion	[25]
Rad53	K22, K213	???	Rpd3	Regulation of kinase activation	[26]
Ume6	K736, K737, K745	Gcn5	Rpd3	Inhibition of transactivation	[27, 28]
Yng2	K170	Esa1	Rpd3	Yng2 stability, NuA4 function	[29]
Swi4	K1016, K1066	???	Rpd3	G1 gene expression	[30]
Shs1	Various/Multiple	Esa1	???	Septin localization	[31]
Atg3	K19, K48	Esa1	Rpd3	Autophagy regulation	[32]

*Note*: Table is a list of non-histone-acetylated yeast proteins for which there exist functional analysis of hyper- and/or hypo-acetylated mutants. Excluded from this list are catalytic subunits of KAT complexes shown to autoacetylate.

In this review, we seek to provide an overview of yeast systems biology techniques currently used to match KATs and KDACs to their substrates. We focus on functional genomic and proteomic methods individually before addressing the outstanding issues in the field.

## Functional genomics methods

### Synthetic Lethal Interactions: A window into unanticipated functions

#### Overview

The development of high-throughput Synthetic Lethal (SL)/sick screens for systematic analysis of genetic interactions of a query gene against either the ∼5000 non-essential deletion mutant strain collection, or, more recently, against conditional alleles of essential genes, has revolutionized yeast genetics and been extensively reviewed elsewhere [33, 34]. Both the Synthetic Genetic Array (SGA) methodology [35, 36] based on robotic manipulation of arrayed strains and the quantitative scoring of growth defects of double mutants, and the alternative diploid-based Synthetic Lethality Analysis on Microarray (dSLAM) [37] have been used to screen KAT and KDAC query mutants to uncover their potential biological functions. Traditionally, SL screens uncover hits that function in parallel to query mutants and impinge on common downstream processes.

#### Examples and advantages

Of the confirmed and putative KATs and KDACs, nearly all have been used as queries and screened against the non-essential deletion set using either SL-SGA [38–41] or dSLAM [37, 29]. Presently, only the essential KAT Eco1 and the putative KATs Lys21 and Spt10 have yet to be used as query genes, although mutants of these KATs have been identified in other SL screens. Genetic interactions from these studies can be accessed and are well annotated in the *Saccharomyces Genome* Database (SGD; yeastgenome.org) [8], the BioGRID (thebiogrid.org) [42] and DryGin (drygin.ccbr.utoronto.ca) [43] databases. While some KAT mutants have only a small number of genetic interactions, like Hat1 with 26, others like Rpd3, Hda1, Esa1, Gcn5 and Rtt109 have over 300 genetic interactions each, with a significant proportion of their genetic network enriched for genes with Gene Ontology annotations assigned to non-nuclear processes. These individual KAT/KDAC genetic interactions offer unprecedented insight into the broader biological functions of these chromatin remodellers. For example, NuA4 mutants display genetic interactions with vesicle-mediated transport genes [29, 41], and, as predicted, these mutants display defects in vacuolar morphology [41].

An advantage of SL screens is that they can be applied to genes encoding all proteins within KAT and KDAC complexes to elucidate the function of various subcomplexes, which would be expected to have similar genetic interaction profiles [44]. For example, hierarchical clustering of NuA4 mutants’ genome-wide genetic interaction patterns uncovered the functional association of Eaf3, Eaf5 and Eaf7 subunits [29, 41], which were subsequently shown to form a submodule of NuA4 [45]. As predicted by the genetic and chemical profiles of *EAF3*, *EAF5* and *EAF7* deletion mutants, a portion of this trimer complex is found outside of the NuA4 complex and has independent functions in modulating chromatin structure and transcription elongation [45]. Because of its novel molecular functions independent of NuA4, this subcomplex has been named the TINTIN complex, for Trimer Independent of NuA4 involved in Transcription Interactions with Nucleosomes [46, 47].

#### Caveats

While SL analysis enables identification of ‘buffering' genetic interactions and identifies potential cellular roles for KATs and KDACs, the nature of the screen does not identify downstream targets or substrates. Further, as KAT and KDACs are functionally redundant for a subset of their cellular roles [29], SL screens likely underestimate the biological processes that individual KAT and KDACs regulate. Another caveat is the majority of SL-SGA screens have been conducted under standard laboratory growth conditions, likely missing genetic interactions that would only be identified under specific conditions like DNA damage or starvation. Finally, because a number of KAT and KDAC enzymes have significant growth defects, there is the risk that the SGA process itself will select for random suppressor mutations that improve growth. Using a system that allows for rapid inhibition of gene expression only after selection of haploid cells with other relevant markers, such as the tet-repressor system that has been used for the screening of essential genes [48], should permit the generation of cleaner SL profiles for these slow-growing mutants.

### Synthetic Dosage Lethality Interactions: Shifting focus to substrates

#### Overview

**Figure 1:**
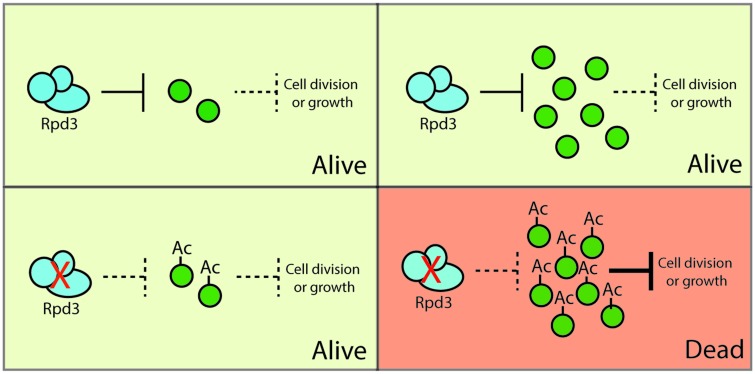
Synthetic Dosage Lethality: Overexpression of substrates can cause lethality in KAT and KDAC mutants via a variety of mechanisms. In the example shown here, Rpd3 functions as a negative regulator of a substrate that is itself a negative regulator of an essential cellular function. Combining overexpression of the substrate with a loss of Rpd3 function results in a loss of cellular viability that is not seen in presence of either single perturbation. (A colour version of this figure is available online at: http://bfg.oxfordjournals.org)

In the Synthetic Dosage Lethality (SDL) technique [33, 49], the SGA approach has been adapted to screen gene overexpression libraries instead of loss-of-function mutations. This allows for identification of both SDL and suppressors of KAT and KDAC mutant queries. Similar to SL-SGA, overexpression of genes can cause the perturbation of complexes and/or hyperactivation of competing pathways, and interactions identified in SDL screens can provide important insight into the biological roles of KATs and KDACs. However, unlike SL-SGA, SDL-SGA screens have also proven successful at identifying downstream targets of signalling pathways, including kinases [50–53], ubiquitin binding proteins [54], KATs [31] and KDACs [30] (Figure 1). Though originally performed using robotic-based array technology, the increasing number of molecular-barcoded open reading frame (ORF) collections and next-generation sequencing approaches is opening the door to screening using a variety of libraries and conditions [55, 56].

#### Examples and advantages

SDL screens have been performed for *HDA1*, *HOS1*, *HOS2*, *HOS3* and *RPD3* KDAC deletion mutants [30] and for non-essential subunits of the KAT complex, NuA4, *EAF1*, *EAF3*, *EAF5*, *EAF6*, *EAF7* [31]. Paralleling their SL/sick genetic interactions, single mutants with growth defects, *rpd3Δ*, *hda1Δ* and *eaf1Δ*, identified the largest number of SDL interactions. As has been seen for kinase [49] and kinetochore [57] query mutants, there is limited overlap in genes identified in SL and SDL screens when the same KAT or KDAC query mutant is used [31]. In addition to using SDL to uncover a novel role for the HDA complex in peroxisome protein import, Kaluarachchi Duffy *et al*. [30] determined that 40% of proteins that are toxic to *rpd3*Δ cells when overexpressed are acetylated *in vivo*, indicating that the *RPD3*-SDL network is enriched for acetylated proteins. Although the acetylation status of all 72 acetylated proteins was not systematically confirmed to be dependent on Rpd3, the acetylation status of the G1 transcription factor Swi4 was. The authors elegantly showed that in *rpd3*Δ cells, the acetylation of Swi4 is increased, resulting in increased binding between Swi4 and Swi6, and activation of Swi4-/Swi6-dependent cell-cycle regulated transcription. Similarly, the SDL screen of NuA4 deletion mutants and related follow-up work revealed a role for NuA4 in septin dynamics and demonstrated that four of five septin proteins are acetylated *in vivo*, that the acetylation state of the Shs1 and Cdc10 septins are partially dependent on NuA4 *in vivo* and that at least Shs1 is a target of NuA4 *in vitro* [31]. Moreover, an Shs1 mutant protein with decreased acetylation *in vivo* displayed septin localization defects similar to those of NuA4 mutants. These two SDL screens illustrate the potential use of SDL in identifying KAT/KDAC substrates.

#### Caveats

While the SDL-SGA has been proven to identify targets of KATs and KDACs, within the individual KAT or KDAC SDL genetic networks, the fraction of proteins that are direct substrates of the queried enzymes remains a mystery. Moreover, this technique does not identify specific sites of acetylation. Hence, though the SDL screen has the ability to identify substrates, significant follow-up study is required to establish bona fide targets and discern the biological consequence of acetylation. A final consideration is the selection of the library to use for screening. Specifically, the two published KAT and KDAC SDL screens used the *GAL1/10*-inducible GST-fusion ORF collection on a multi-copy plasmid [58]. With a growing role for acetylation in regulating carbon metabolism in yeast [7, 16], the utilization of galactose may prove to be less than ideal, and the use of other overexpression systems should be considered.

### High-Content Screening— Uncovering the role of KATs and KDACs in vivo

#### Overview

The development of high-throughput microscopy and the ORF-GFP collection [59] has allowed for the systematic screening of protein abundance and localization [reviewed in 60]. High-content screening (HCS) of the ORF-GFP collection under a variety of environmental stress or genetic perturbations provides a systems-level view of proteome dynamics [61–63]. In an alternative approach, SGA technology can be applied to combine a mutant collection with a specific fluorescently tagged protein(s), with the goal of ascertaining which pathways impact the localization of specific proteins/organelles such as tubulin [64] or P-bodies [65]. With recent advancements in machine learning approaches to systematically quantitate localization and protein abundance levels [62], there is likely to be significant pay-off in using HCS to study KAT and KDAC functions.

#### Examples and advantages

Although cellular compartmentalization may define KAT and KDAC substrate targeting**,** surprisingly little is known about the localization of these proteins. A few clues are starting to emerge, however, indicating that KATs and KDACs might be more dynamic than originally thought. For example, in directed studies it was found that Esa1 and Rpd3 transiently localize to pre-autophagosomal structures on nitrogen starvation [32], and Hos2 and Hst2 localize to cytoplasmic granules during stationary phase [66]. In HCS studies, it was demonstrated that Hos2-GFP localizes to nuclear Cmr1-containing foci during DNA damage [63], and that Hos1-GFP localizes to cytoplasmic foci on nitrogen starvation [61]. As more conditions are tested and entered into searchable databases such as CYCLoPS (http://cyclops.ccbr.utoronto.ca/) [67], LoQAtE (http://www.weizmann.ac.il/molgen/loqate/) [68] and the Yeast Resource Centre Image Repository (http://images.yeastrc.org/), a more comprehensive picture of KAT and KDAC dynamics within the yeast cell will undoubtedly emerge.

It is also becoming clear that KATs and KDACs impact localization and function of a wide variety of proteins. For example, screening for aberrant spindle morphology across the deletion mutation collection revealed that deletion mutants of *RTT109*, *ELP3* and numerous non-essential mutants of the NuA4 KAT complex, have defects in various aspects of spindle dynamics [64]. Subsequent studies identified protein co-purification between NuA4 and spindle pole body proteins and confirmed a role for NuA4 in spindle dynamics, although the mechanism involved remains unknown [69]. Using an inverse approach, *rpd3Δ* has been mated to both a mini-array [30] and the entire ORF-GFP collection [62]. Both of these studies revealed that Rpd3 regulates the cellular localization of a diverse range of proteins, and at least a subset of these proteins is hyperacetylated in *rpd3Δ* acetylome studies. As such, it has been hypothesized, but not proven, that the acetylation state of these factors may directly regulate their localization.

#### Caveats

Presently the largest caveats to this approach are access to high-throughput microscopy platforms that have the ability to perform live imaging with quick changes in conditions (e.g. media, temperature), and the ability to quantitate phenotypes. Further, like other functional genomic screens, these screens can identify potential novel cellular roles for KATs and KDACs, but do not identify the molecular mechanism or substrate(s) at play.

## Proteomics methods

### Affinity purification of KAT and KDACS: Building networks

#### Overview

KAT or KDACS are immunoprecipitated from cell extracts, and interacting proteins are digested with site-specific proteases to allow detection via mass spectrometry (IP-MS).

#### Examples and advantages

Examples of directed IP-MS-derived ‘interactomes’ for KAT and KDACs are numerous. Interaction partners have been extensively catalogued through a number of proteome-wide interaction studies for all KAT and KDACs (including both IP-MS and two-hybrid approaches) [70–72]. Data from both low- and high-throughput experiments are well annotated in the SGD and the BioGrid databases. Using the toolset available at SGD, interactors can be filtered by the number and type of experiments in which they were observed. In addition, this methodology may provide insight into the role of individual subunits within multi-protein KATs. For example, the Washburn group purified the SAGA complex containing the Gcn5 KAT in various SAGA subunit deletion mutant backgrounds to define submodules and the overall architecture of the complex [73]. In addition to full deletions, utilization of domain deletions, point mutants and cross-linking reagents combined with IP-MS have further defined the structure and subunit interconnectivity of SAGA [74–76].

#### Caveats

A caveat of co-interacting proteins experiments is that substrates may interact with KAT or KDAC enzymes only transiently, precluding their recovery. Even stoichiometric binding partners may not be direct targets, even if they are acetylated. Before such claims can be made, detailed follow-up, including a comparison of acetylations on candidate targets recovered from wild-type and mutant cells, and, ultimately, *in vitro* (de)acetylation assays should be done. As such, what these studies may provide, more than information on targets *per se*, is a window into unanticipated functions of individual KAT and KDAC enzymes that can be inferred from unique (non-substrate) interactors.

### Protein chips: Dusting off microarray technology

#### Overview

GST-fusion proteins purified from yeast [58] are attached to a slide that is overlaid with purified KAT enzyme (either alone or as members of larger complexes) and radiolabelled acetyl-CoA. After allowing for acetylation of fusion proteins, the slide is washed and imaged to identify *in vitro* KAT targets [16].

#### Examples and advantages

The utility of this approach was demonstrated by Zhu and colleagues, who used proteome chips to identify *in vitro* substrates for the NuA4 complex [16]. Because this technique profiles the entire proteome without the preconceived notion of enzyme function, it is well suited to the discovery of unanticipated substrates of KAT enzymes spanning a variety of functional categories. For example, follow-up studies demonstrated that NuA4-dependent acetylation of two identified targets, Pck1 [16] and Sip2 [17], is important for the regulation of chronological and replicative lifespan, respectively.

#### Caveats

Substrate identification using proteome chips depends on the ability to purify a KAT enzyme in an active form and with complex members that facilitate its activity or dictate targeting specificity *in vivo*. Similarly, correct folding and/or the presence of other interactors of chip-bound substrates may be required for KAT binding. On the other hand, some proteins that never encounter a given KAT in an *in vivo* setting may nevertheless suffice as substrates *in vitro*.

### αAck IP-MS (‘Acetylome Profiling’): Going big

#### Overview

**Figure 2:**
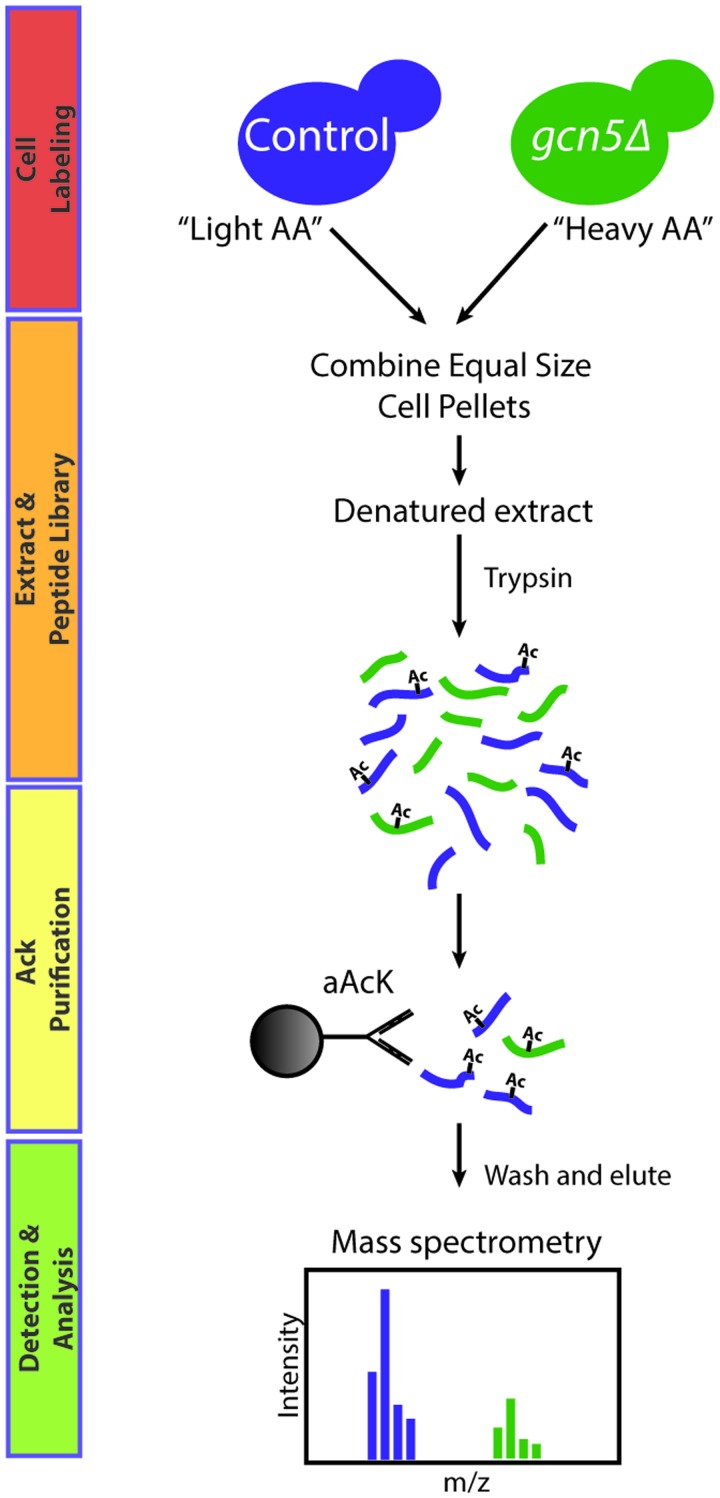
Acetylome profiling: Wild-type and mutant cells are grown in the presence of light combination of cell pellets and denaturing lysis. A peptide library containing both heavy- and light-labelled peptides is generated by cleaving with trypsin or another site-specific protease, and acetylated species are purified with an antibody against acetylated lysine. After washing and elution, peptides are analysed by MS. Acetylated species are detected as paired peaks with the intensities reflecting abundance within the cell. For KAT mutants (shown here), the heavy to light ratio is expected to be <1 for regulated peptides. For KDAC mutants, regulated sites are found on peptides with a heavy:light ratio of >1. (A colour version of this figure is available online at: http://bfg.oxfordjournals.org)

In this technique [4, 6], antibodies that recognize acetyllysine residues within a variety of different amino-acid sequences are used to enrich for acetylated peptides following cleavage of whole-cell extracts with trypsin or other site-specific proteases. Following peptide elution, acetylation sites on recovered peptides are identified using tandem mass spectrometry (MS). In a variation of this technique, *s*table *i*sotope *l*abelling of *a*mino acids in *c*ell culture (SILAC) is used to differentially label wild-type cells and those mutated for a given KAT or KDAC of interest (Figure 2). This allows for the generation of a single internally controlled protein extract that can be used to generate a library containing acetylated peptides from both starting strains. Following immunoprecipitation and MS analysis, acetylated peptides are detected as paired ‘heavy’ and ‘light’ peaks. The ratio of peak intensities is representative of the prevalence of a given acetylation in one strain versus another.

#### Examples and advantages

With the earlier proteome-wide identification of acetylation sites described for bacteria (2010) [77], drosophila (2011) [5], mouse (2006) [78] and human cells (2009) [79], it is perhaps surprising that the first ‘acetylome’ analysis for *S. cerevisiae* was not unveiled until 2012 [4]. When published, this work confirmed what most researchers had suspected—that in yeast too, lysine acetylation is a pervasive modification, targeting proteins with diverse biological functions

Acetylome profiling has been carried out for cells lacking the individual KDACs, Rpd3 [4], Hst2, Hst4 and Sir2 [80], and for a triple KDAC mutant (*hst1Δ hst2Δ sir2Δ*) [6]. In addition, three different KAT enzymes, Gcn5, Esa1 and Hat1, have been profiled [6]. What has emerged from these analyses—beyond lists of individual acetylation sites—is evidence for multiple KATs and KDACs functioning in a broad range of cellular processes. Analysis of *gcn5Δ* cells, for example, uncovered Gcn5-regulated acetylations on 34 high-confidence proteins with roles in chromatin biology and transcription (as might be expected for a nuclear KAT), but also in ribosomal RNA processing and ribosome biogenesis. Bioinformatic analysis of regulated acetylation sites also allows for the generation of consensus sequences. For example, Downey *et al.* [6] found that Gcn5 and sirtuin KDAC enzymes tend to regulate lysine residues surrounded by a similar set of amino acids, and these sequences are distinct from those regulated by Esa1. Previously, a number of groups have used predictive approaches to identify new acetylated proteins in yeast based on sequences surrounding acetylated lysine residues on known targets [81, 82]. The continued integration of large-scale data sets into these programs should only improve their predictive power. It will also be interesting to see if *in silico* analyses can uncover motifs, distinct from modified lysines themselves, on substrates of individual KATs or KDACs that facilitate enzyme–target interactions or suggest downstream functions for dynamically regulated acetylations.

#### A window into acetylation stoichiometry

An important contribution from Weinert *et al*. [7] combined SILAC and acetylome profiling with chemical treatment of light-labelled cultures with acetylphosphate—which non-enzymatically acetylates lysine residues that are not already acetylated at a low (but defined) level. Comparison of heavy to light ratios allows for the identification of proteins having lysines that are frequently acetylated, as opposed to minor acetylation sites [7]. Using this method, the authors found that high levels of acetylation are the exception rather than the rule, with the greatest levels occurring on nuclear proteins [7]. As discussed below, it is likely that other proteins show increased stoichiometry of acetylation under specific conditions.

#### Caveats

While the ‘acetylome profiling’ technique is well suited to the identification of sites regulated by both KATs and KDACs, there is no guarantee that such sites are direct targets. Moreover, critical acetylations regulated redundantly by multiple enzymes may appear unchanged in acetylomes derived from single mutants, and analysis of double mutants may not always be possible because of SL relationships between KATs or KDACs having overlapping functions.

Another drawback of this technique is its dependence on αAcK antibodies—which may be subject to significant variation in sequence preference from source to source, or even lot to lot. As such, targets recovered and consensus sequences derived from this method may give an incomplete picture of enzyme targeting and function. The use of different antibodies, either separately or as a mix, may improve the scope of future acetylome analyses. Alternatively, Bryson *et al*. [83] recently demonstrated that bacterially expressed acetyllysine-binding bromodomains can be used to enrich for acetylated species in acetylome studies. A final consideration—relevant to all techniques using MS to identify acetylation sites—is that acetylated lysine residues are nearly isobaric to trimethylated lysine. As such, these two modifications can be difficult to differentiate from one another, and proper analysis requires the use of mass spectrometers having high mass resolving capabilities.

### mCHIP-KAT-MS: The best of both worlds?

#### Overview

This technique [69] introduces a clever twist to standard IP-MS analysis used for interactome studies. Following KAT immunoprecipitation, the co-immunoprecipitated material is incubated with heavy-labelled acetyl-CoA and stringently purified KAT complex. After incubation under conditions permissive for *in vitro* acetylation, the purified proteins are analysed to identify KAT interactors, *in vivo* acetylation sites as well as *in vitro* catalyzed acetylation sites—which can be identified by the mass shift imparted by the heavy-labelled acetyl group.

#### Example and advantages

The strength of this technique lies in its ability to identify putative acetylation sites targeted both *in vivo* and *in vitro* by a KAT enzyme of interest. For example, Mitchell *et al*. [69] used mCHIP-KAT-MS to identify 66 acetyllysine residues on 23 proteins that co-purified with Esa1-TAP, of which 6 acetyl lysine peptides on 5 proteins were detected with both *in vivo* (light) and *in vitro* (heavy) acetyl groups. These sites represent strong candidates for NuA4-target acetylation sites, as they occur *in vivo* and NuA4 can catalyze the reaction. A second strength of this technique is that it does not rely on antibody-based recovery of acetylated proteins, which, as discussed above, can be heavily influenced by the antibody source.

#### Caveats

A disadvantage of this technique is that it does not determine if a given acetylation site is also targeted by other KAT enzymes or the proportion of the protein population that is acetylated. A second caveat of this technique is that it relies on sustained, albeit not necessarily direct, interaction between the KAT complex and substrate for recovery of both *in vivo* and *in vitro* catalyzed acetylations. Many substrates may instead interact only transiently with their cognate KATs, as is often the case for enzyme–substrate pairs. A potential solution here may be to treat cells used for KAT purification with chemical cross-linkers, which may preserve transient interactions. Application of the newly described BioID technique [84], which allows for *in vivo* biotinylation and streptavidin recovery of transient interactors should also work to increase the scope of the mCHIP-KAT-MS technique. An alternative solution is to perform an inverse mChIP-KAT-MS, in which bait proteins purified by mChIP are used in a KAT assay with heavy-labelled acetyl-CoA before MS. Finally, as with other MS-based techniques, there is no guarantee that identified acetylation sites will be visualized by low-throughput techniques such as Western blotting, which can stifle the follow-up of individual target proteins.

### Biotinylation of Lysine Method: Do not forget about the KDACS

#### Overview

This method [85] uses chemical acetylation to quantitatively acetylate all available lysine residues before carrying out deacetylation reactions using purified KDAC enzymes. A biotinylation reaction is then used to attach biotin to deacetylated lysines, before purification with streptavidin-coated beads. Subsequently, biotinylated residues can be identified via MS.

#### Example and advantages

While the proteome chip and mChIP-KAT-MS techniques described above are well suited for the large-scale analysis of *in vitro* acetylations catalyzed by KAT enzymes, they provide no information on the KDACs responsible for reversing those acetylations. As a counterpart to those techniques, the biotinylation of lysine method provides a window into KDAC specificity and function. Bheda *et al*. [85] used this method to identify histone H3 lysine 79 as a direct substrate of yeast Sir2. As noted previously [85], this method should also be useful for studying the role of KDACS in removing other acylations (see below).

#### Caveats

Because this is necessarily an *in vitro* technique, it is subject to the same biases as *in vitro* assays geared towards deciphering KAT function: Identified protein substrates may never be encountered *in vivo*, and purified KDACs may display altered specificity in the context of *in vivo* binding partners (either characterized or uncharacterized).

## Implications and future directions:

### Combining techniques: Where the rivers meet there is gold

Of all the KAT and KDAC enzymes, the essential Esa1 KAT of the NuA4 complex has been studied using a wide variety of different techniques, both genetic and proteomic. As such, data sets emerging from these screens can be analysed for both similarities and differences. Comparing techniques most likely to identify substrates—high- and medium-confidence Esa1-regulated acetylations from an acetylome study [6], Esa1-dependent *in vitro* substrates from protein arrays [16], *in vitro* targets and protein interactions identified in Esa1 mChIP-KAT-MS [69] and NuA4 SDL interactions [31]—the overlap is remarkably slim. Despite nearly 300 proteins identified in these screens, with the exception of NuA4 complex subunits and histone proteins, only 8 proteins were identified in two or more distinct screens. These include Spt6 [6, 69], Cnm67, Rpl8a [16, 69], Msn4, Shs1, Rpl4a [31, 69], Gsy2 and Whi5 [16, 31]. Though the overlap is low, at the cross-section are proteins whose biological functions are linked to NuA4, including Spt6 [86], Msn4 [41, 87, 88] and Shs1 [31]. Hence, as the body of knowledge surrounding KATs and KDACs continues to grow, the field will benefit from the integration of acetylation data (regulated sites, responsible KATs, KDACs, conservation, etc.) into searchable databases such as SGD and BioGrid.

### Cross-talk and competition: Whose on first?

Connecting individual acetylation sites to specific phenotypes is complicated by a wealth of other lysine-based modifications including sumoylation, ubiquitylation and methylation, whose downstream signalling processes can also be disrupted by the substitution of target lysine residues for arginine or glutamine in mutant proteins. Moreover, a number of recent studies highlight the potential importance of other acyl-chain-based modifications—butyrylation [89, 90], propionylation [89], succinylation [90, 91] and crotonylation [92]. Intriguingly, these modifications could be regulated by the same enzymes responsible for acetylation and deacetylation reactions. For example, work from the Denu group has demonstrated that Hst2 binds to propionylated peptides with greater affinity and exhibits greater deacylation of those peptides than for acetylated peptides [93, 94]. More recently, Sabari *et al*. found that p300 (a structural ortholog of the yeast Rtt109 KAT [95]) can catalyze crotonylation of histone H3 on lysine 18 and that this modification regulates transcriptional output [92]. With these data in mind, it is clear that acetylation—or any PTM for that matter—cannot be studied in isolation. Integration of ‘acetylome’ data sets with those generated for other modifications (both lysine and non-lysine based) should provide a resource to interpret experiments aimed at connecting acetylation sites to particular phenotypes. In a similar vein, it will be important to determine how acetylation and other lysine based modifications regulate each other in space and time—on the same residue through direct competition or on neighbouring residues within the same domain or motif.

### Expanding Conditions: The KATs (and KDACs) are stressed out

Not only is there evidence of KAT and KDAC enzymes relocalizing on stress (see above), but dramatic changes in the acetylome occur on changes in metabolism [7]. Therefore, it is imperative that systems-level approaches start addressing how KATs and KDACs remodel the proteome under various conditions. One approach may be to exploit a combination of live-cell HCS with acetylome profiling and other proteomic approaches under various stress conditions. Not only will this approach allow for the identification of how KATs and KDACS locate their substrates under specific circumstances, but it will also provide information about which of those targets have significant changes in localization or protein abundance. Such data, which will pinpoint biologically relevant KAT/KDAC–substrate relationships, can then be used to prioritize targets for more detailed follow-up analyses.

#### Final Remarks

The research community has now moved beyond the simplistic view of acetylation as a PTM regulating histones and affecting gene transcription. Undoubtedly, the yeast model system is well positioned to take the lead, as we move from identifying acetylated proteins to connecting specific KATs, KDACs and their target sites to meaningful biological functions. Integration of emerging technologies into the repertoire of techniques used to study acetylation will ensure that the yeast community can continue to make important contributions to this burgeoning field.

Key Points

- Acetylation is a common post-translational modification, but few KATs and KDACs have been matched with their substrates, and little is known about the functions of individual acetylations.
- Functional genomic techniques and high-content imaging give insight into pathways regulated by KAT and KDAC enzymes.
- Proteomic techniques provide details on KAT's and KDAC's complex architecture and substrate selection.
- A combination of techniques is necessary to understand how KATs and KDACs regulate important processes within the cell both alone and in concert with other post-translational modifications

## Funding

This work was supported by grants from the Natural Sciences and Engineering Research Council of Canada (RGPIN-326770) and Canadian Institutes of Health Research (MOP-142403) to K.B. and start-up funds from the University of Ottawa to M.D. K.B. is a Canada Research Chair in Functional and Chemical Biology. We thank Michael Kennedy for critical reading and suggestions. We apologize to those colleagues whose work we did not cite because of lack of space.
